# Effects of Culture Systems and Feed Types on Water Quality and Growth Performance of Japanese Eel (*Anguilla japonica*)

**DOI:** 10.3390/ani15162420

**Published:** 2025-08-18

**Authors:** Jimin Choi, Ju-ae Hwang, Hyeong Su Kim, Jeonghwan Park

**Affiliations:** 1 Advanced Aquaculture Research, National Institute of Fisheries Science, Changwon 51688, Republic of Korea; jmcchoi@naver.com (J.C.); hjuae1031@korea.kr (J.-a.H.); 2 Aquaculture Research Division, National Institute of Fisheries Science, Busan 48513, Republic of Korea; 3 Department of Fisheries Biology, Pukyong National University, Busan 48513, Republic of Korea

**Keywords:** recirculating aquaculture system, feed type, water quality, growth, stress biomarkers

## Abstract

Utilizing paste feed within a recirculating aquaculture system for eel cultivation may lead to various challenges, such as water quality degradation and, hence, transitioning to floating pellet type feed becomes essential. In this study, we aimed to investigate the impact of different feed types (paste and floating extruded pellet) and rearing systems (recirculating aquaculture and flow through system) on water quality, growth, blood chemistry, body composition, and expression levels of digestive enzymes in Japanese eels (*Anguilla japonica*). Throughout the experiment, notable variations were observed in total ammonia nitrogen and nitrite nitrogen levels, with higher concentrations in the recirculating aquaculture system (1.00 ± 0.64 and 0.757 ± 0.464 mg/L, respectively) than paste groups (0.859 ± 0.651 and 0.485 ± 0.502 mg/L, respectively). Significant differences were observed in weight gain and specific growth rates, with the pellet group (154 ± 10% and 1.50 ± 0.06%/day, respectively) exhibiting higher values than the paste group (135 ± 13% and 1.37 ± 0.09%/day, respectively). Blood parameters showed significant differences depending on the culture system, with generally higher values observed in the flow-through system, while no significant differences were observed between feed types. Whole-body composition exhibited variations attributed to feed intake, with notable differences in crude protein and crude fat content among the experimental groups. The expression levels of digestive enzymes and nutrient transporters were higher when the eels were fed pellet-type feed and reared in recirculating aquaculture system. The study findings indicate that pellet feed enhances water quality management in RAS, leading to improved eel growth. Given its lower environmental stress, pellet feed is preferable to paste feed for optimizing eel production in RAS.

## Simple Summary

Despite its widespread use in eel aquaculture, paste feed can cause significant water quality issues in recirculating aquaculture systems (RAS) due to its tendency to break down easily in water. In contrast, floating extruded pellets are more water-stable and enable better control over feeding practices. This study evaluated the effects of feeding different feed types (paste type and floating pellet type) and different culture systems (RAS and flow-through system) on water quality, growth performance, blood chemistry, and digestive enzyme expression in Japanese eel (*Anguilla japonica*). The results showed that floating pellet feed contributed to improved water quality and growth in RAS. These findings indicate that floating pellets are a more suitable feed type for use in RAS, and can support more effective and sustainable management in eel farming operations.

## 1. Introduction

The Japanese eel (*Anguilla japonica)* is a key species in Korea’s inland aquaculture, valued for its economic and nutritional benefits. Notably, eels have contributed significantly to this production surge, ranging from 11,000 to 18,000 tons, and constituting approximately 30% of the output of Korea’s inland fisheries. The economic importance of eel farming is underscored by its position as the second-highest production value species in Korea, with a value of KRW 511,144 million, following flatfish with a value of KRW 703,438 million [1]. 

Freshwater eel, particularly Japanese eel (*A. japonica*), is a key species in Korea’s inland aquaculture, valued for its economic and nutritional benefits. It is recognized as a high-value-added fish due to its rich content of protein, collagen, and vitamins, and its reputation as a nutritious food has contributed to its continued popularity [2]. According to Schreckenbach et al. [3], European eel (*Anguilla anguilla*), a closely related species, showed the highest lipid content and energy density among 17 freshwater fish species, indicating the high nutritional potential of eel. In addition, among four farmed eel species evaluated in Korea, *A. japonica* received the highest scores for taste, flavor, and overall preference in a sensory test [4], supporting its status as a premium aquaculture product.

Many eel farming practices have historically employed intensive pond culture systems; however, in Korea, a recent shift towards high-density recirculating aquaculture systems (RAS) has been observed, with the aim of improving productivity and reducing production costs in eel farming [5]. The RAS stands out as a prominent ecofriendly aquaculture method, characterized by reduced water consumption through the recirculation of breeding water, resulting in a diminished environmental impact through effective effluent management and nutrient recycling [6,7]. Despite its high initial and management costs, RAS minimizes environmental impact and supports high-density farming with stable temperatures. Its shorter growth period and higher productivity are driving a shift from conventional farming to RAS for improved efficiency and reduced costs [8].

Feed selection is crucial in aquaculture management [9], particularly in eel farming, where paste feed is predominantly used, while only a few farms opt for floating extruded pellets [10]. However, paste feed disintegrates easily, which may lead to significant loss, increased suspended solids, and water quality deterioration in RAS [11,12]. Its rapid breakdown generates solid waste, disrupts filtration, and elevates the risk of system failure. Accumulated solids stress eel gills, hinder growth, and promote pathogen proliferation, increasing susceptibility to infection [13,14,15]. Dissolved solids from paste feed fuels microbial activity, further degrading water quality. Excess solids stimulate heterotrophic bacterial growth, depleting dissolved oxygen and increasing CO_2_, negatively impacting eel metabolism. The accumulation of solids and waste interferes with autotrophic bacteria in biological filtration, disrupting nitrification and raising ammonia and nitrite levels. Increased turbidity reduces sterilization efficiency, heightening disease risks. These issues compromise the functionality of filters, sterilizers, and pumps, leading to frequent maintenance and increased costs [16,17].

In contrast, all the aforementioned negative impact of paste feeding related to an increased amount of solid waste can be overcome by floating extruded feed. Furthermore, utilizing floating extruded pellet enables farmed animals to access their feed from the water surface, affording fish farmers the opportunity to observe the feeding responses of cultivated fish and tailor the feeding regimen accordingly [9,11,12]. Consequently, a transition from paste feed to floating extruded pellet is deemed imperative for the steadfast management of aquaculture systems and for augmentation of production when implementing RAS in eel farming.

The performance of recirculating aquaculture systems (RAS) is known to be influenced by multiple factors, including water temperature, stocking density, water quality parameters, and system design and operation [15]. Among these, feed type remains an important variable, especially in relation to system management and fish performance under different culture conditions. Previous studies have compared extruded pellet and paste diets in Japanese eel (*A. japonica*) and reported improved growth and water quality in pellet-fed groups under flow-through conditions [10]. However, the interaction between feed type and different culture systems, such as RAS, has not been fully explored. Therefore, this study assessed the impact of feed type (paste vs. floating extruded pellet), culture system (FTS vs. RAS) and their interaction on water quality, eel performance, health and stress parameters. Through these investigations, our goal was to affirm the suitability of employing floating pellet feed in RAS for eel farming, while concurrently gathering fundamental data essential for formulating management strategies in the operation of an ecofriendly eel farm.

## 2. Materials and Methods

### 2.1. Experimental Conditions

#### 2.1.1. Sample Collection and Ethical Considerations

Sample collection adhered to the Guidelines for Experimental Animals of the National Institute of Fisheries Science (NIFS) Institutional Animal Care and Use Committee (2023-NIFS-IACUC-25) and was conducted at the Advanced Aquaculture Research Center Jinhae, Republic of Korea, affiliated with the NIFS.

#### 2.1.2. Fish and Environment Conditions

Juvenile eels were purchased from a local eel farm (Saeng-ginala farm, Icheon, Republic of Korea) and transferred the Advanced Aquaculture Research Center of the National Institute of Fisheries Science. During a two-week acclimatization period, the eels were gradually accustomed to the experimental tank conditions, including water temperature, pH, and DO, to minimize stress. Randomly selected eels, with an average total length of 326 ± 22 mm (means ± S.D.) and an average weight of 32.1 ± 0.4 g, were used in the experiments. The study was conducted in a 2 × 2 factorial design with two different systems (flow through systems (FTS) and RAS) and two different feed types (paste and floating extruded pellet), in triplicate. The experimental setup consisted of 12 individual systems, including 6 FTS and 6 RAS tanks. Each tank housed an average of 62.5 ± 0.9 eels, totaling 2.00 ± 0.02 kg in biomass, with a stocking density of 5.01 ± 0.04 kg/m^3^ per tank. The overall total weight and head count were equalized using values incorporating the standard deviation of the mean weight. The experiment commenced after a one-week acclimatization period in the tanks assigned to each experimental group for adaptation to the respective feeding regimes and the environment. During this period, the eels were fed 1% of their body weight for six days with the corresponding feed assigned to each experimental group and were fasted for 24 h before the start of the trial. No stress-related behaviors or mortalities were observed during the acclimatization period.

#### 2.1.3. Feeding Regimen

During the 62-day experimental period, eels were provided with either paste or floating extruded pellet diets in FTS and RAS, respectively, with each treatment conducted in triplicate. In the case of paste feed, there was difficulty in manufacturing paste feed and floating extruded pellet with the same ingredients due to the addition of starch for viscosity when kneading. We attempted to manufacture feed for the experiment, but, because of this, we chose feed that is widely used in eel farming sites. Commercially available powdered feed (Black powder, Suhyup, Uiryeong, Republic of Korea) and floating extruded pellet (FGR, Purina, Seongnam, Republic of Korea) were utilized, with proximate feed analysis detailed in Table 1. The feed composition was analyzed according to the methods described by the Association of Official Analytical Chemists (AOAC) [18]. Feed was administered twice daily (at 10:00 and 17:00 h) to ensure satiation. Paste feed was made by mixing powdered feed with water in a 1:1 ratio. Groundwater was used for mixing, filtered through a 5 μm microfilter (PP filter, Human Science Co., Ltd., Hanam, Republic of Korea) prior to use. Both pellet and paste feeds were weighed before and after feeding. To ensure consistency in nutrient input, the exact amount of feed provided was recorded, and uneaten feed was collected, dried, and weighed. Feeding activity was monitored under low-intensity red lighting to minimize stress on the fish. Remaining feed was retrieved using a flashlight, then measured at a separate location.

#### 2.1.4. Environmental Parameters

In RAS, water temperature was maintained constant at 25 °C by using a 1 kW heater (OKE-HE-100, Sewon OKE, Busan, Republic of Korea) whereas, in FTS, the system experienced temperature variations due to low-temperature inflow water (approximately 15–16 °C). To minimize fluctuations in FTS, a 2kW heater was employed. An air heater (RNW2900P2S, LG Electronics, Seoul, Republic of Korea) was used to maintain the air temperature in the tank room at 25 °C. The DO levels (>6 mg/L) were ensured by installing two air stones in each tank, powered by an air blower (Hiblow HP-200, Techno Takatsuki Co., Ltd., Osaka, Japan). The inflow water to the FTS had a pH of approximately 6.47 ± 0.08. The pH levels in the RAS experimental tanks were intermittently adjusted based on the FTS experimental zone. If the pH levels in the RAS dropped below these levels during water quality measurements, sodium bicarbonate (NaHCO_3_, OCI Company, Seoul, Republic of Korea) was dissolved in distilled water and added as needed until the end of the experiment. The photoperiod was maintained at 24 h of darkness, as is the practice in aquaculture farms. The experimental setup included triplicate systems for both RAS and FTS conditions, ensuring consistent environmental parameters across all replicates.

#### 2.1.5. Water Management

The 62-day experiment was conducted without water exchange in RAS to observe changes in water quality. While exchanging water would have a positive effect on the water environment for the experimental fish, it would also result in changes in nitrate accumulation and other water quality parameters. This approach was chosen to simulate conditions that might be encountered in commercial-scale RAS, wherein water conservation is a key consideration and the accumulation of substances over time must be managed effectively. Water was added in RAS only to replace the amount lost through evaporation. The water refreshment rate in FTS was set to 1.7 L/min. Groundwater, filtered through a 5 μm microfilter (PP filter, Human Science Co., Ltd., Hanam, Republic of Korea), was utilized in all experimental tanks.

### 2.2. System Configuration

**Figure 1:**
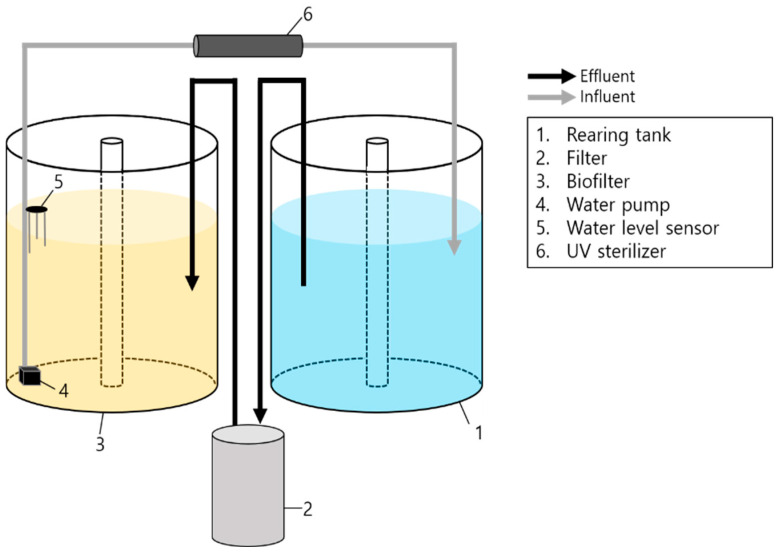
Schematic drawing of the experimental recirculating system used in the experiments.

Six FTS and six RAS were each set up in separate 500 L polypropylene circular tanks (diameter 100 cm × height 50 cm; water volume 0.4 m^3^). The FTS utilized a venturi drainage system, whereas the design of RAS employed in this study is illustrated in Figure 1, which depicts its essential components. The RAS system used in the experiment was designed to function similarly to commercial aquaculture equipment. Each RAS comprised a biological filtration tank, an external filter (20 W, EHEIM classic 600, EHEIM, Deizisau, Denmark), and a UV sterilizer (18 W, UV-A18W, PERIHA, Zhongshan, China). A polynagel sponge (35 ppi, DYnTEC, Seoul, Republic of Korea) was placed inside the external filter to effectively capture the solids. The filter was maintained weekly to ensure optimal performance. This schedule was chosen to balance labor efficiency and system performance. Daily maintenance was considered but found impractical due to resource limitations. Additionally, our trials indicated that weekly cleaning did not significantly impact the water quality or led to substantial leakage and potential release of captured solids. The cleaning cycle was configured to a duration that did not impact the flow rate. The biological filtration tank housed 100 L of fluidized bed filter medium (TK1, TaeJin Engineering, Busan, Republic of Korea) aged for over a month. A union hose facilitated continuous floating of the filtered media. To maintain water circulation, a 15 W pump directed water through the UV sterilizer and back into the rearing tank. An electrode-type automatic water level sensor (YQ-5000, Youngjin, Republic of Korea) was integrated with the pump, ensuring a consistent water level in both the rearing and filtration tanks.

### 2.3. RAS Flow Rate Optimization

To regulate the total ammonia nitrogen (TAN) concentration within the optimal range (≤23 mg/L) [19], the flow rate was optimized, not merely based on the flow itself but, importantly, considering the nitrification efficiency of the biological filter, which is crucial for maintaining safe TAN levels. The dimensions of the biofilters were based on the surface specific area of the media used, which was 500 m^2^/m^3^, and the surface specific nitrification rate, assumed at 0.3 g TAN /m^2^∙day^−1^. These parameters are critical as they directly influence the TAN conversion capacity of the system.

The TAN excreted by eels was computed by considering the protein content in the feed, based on floating extruded pellet. The net protein utilization was set at 20%, as per Jauncey [20]. Equation (1) was employed to ascertain the daily TAN production amount. Subsequently, Equation (2) was utilized to calculate the maximum flow rate capable of handling the daily TAN production volume, ensuring that it aligned with the nitrification capacity of the biofilters. This approach ensured that the flow rate was adjusted according to the nitrification capacity of the biofilters. The TAN concentration in the effluent from the fish tank to the biological filtration tank adhered to the safe TAN concentration for eels. The TAN concentration in the influent flowing back into the rearing tank was established in accordance with the nitrogen treatment capacity of the biological filtration tank within the system.

Consequently, the flow rate of the system was optimized at 1.7 L/min (6.12 rotations/day), aligning with the bio-filer’s ability to maintain the TAN within safe limits for eels. The flow rate for the FTS experimental group was also set to this value to ensure uniform conditions.

(1) $${TAN}_{p}=\left(1-NPU\right)\times P_{f}\times N\times W$$

where,

TAN_p_ = TAN production amount (kg/day),

NPU = Net protein utilization (%),

P_f_ = Protein content in feed (fraction),

N = Nitrogen content in protein (fraction),

W = Feed amount (kg/day).

(2) $$Q=\frac{{TAN}_{p}}{[1440\times \left(C_{out}-C_{in}\right)]/1,000,000}$$

where

Q = Flow rate (L/min)

C_in_ = TAN concentration in influent (mg/L),

C_out_ = TAN concentration in effluent (mg/L),

1440 and 1,000,000 = Conversion units.

### 2.4. Water Quality Analysis

Water quality parameters in the culture water were assessed daily per tank, specifically before the morning feeding, utilizing a water quality meter (YSI-650; Yellow Springs Instruments Inc., Yellow Springs, OH, USA). The measured parameters included water temperature, DO, pH, electrical conductivity (EC), and total dissolved solids (TDS). Additionally, the concentrations of TAN, nitrite nitrogen (NO_2_^−^-N), and nitrate nitrogen (NO_3_^−^-N) were obtained before each feeding session in triplicate. These nitrogen compounds were quantified using an analysis reagent kit (Merck KGaA, Darmstadt, Germany), and water quality analysis, encompassing these parameters, was carried out three times a week employing an absorption photometer (Spectroquant^®^ Prove 100, Merck KGaA, Darmstadt, Germany).

### 2.5. Growth Rate

Upon the conclusion of the experiment and following a 24-h fasting period, 30 eels were randomly selected per tank and were administered immersion anesthesia at a concentration of 100 ppm using MS-222 (Sigma-Aldrich, St. Louis, MO, USA). For consistency, the eels were also fasted for 24 h at the beginning of the trial. Subsequently, the average total length (mm) and weight (g) of the eels were measured. The recorded values were utilized to calculate various growth-related parameters, including the weight gain rate (WGR), specific growth rate (SGR), survival rate, and condition factor (CF) of the experimental fish. Moreover, the internal organ weight (g) and liver weight (g) were measured for five fish per tank, and the viscerosomatic index (VSI) and hepatosomatic index (HSI) were determined through the following formulae:

WGR (%) = [Final weight (g) − initial weight (g) × 100]/initial weight (g)

SGR (%/d) = [Log_e_(final weight (g)) − Log_e_ (initial weight (g))] × 100/days

Survival rate (%) = (Final number of individuals/initial number of individuals) × 100

CF = [wet weight (g)/(total length (cm))^3^] × 100

VSI (%) = [wet weight of viscera (g)/wet weight (g)] × 100

HSI (%) = [wet weight of liver (g)/wet weight (g)] × 100

### 2.6. Blood Parameters

Various blood parameters, including stress hormones, antioxidant enzymes, and liver function enzymes, were analyzed to evaluate the impact of feed type and aquaculture system on stress levels in eels. At the termination of the experiment, blood samples were collected from the caudal vessels of five fish per tank using a 1 mL syringe treated with heparin (Sigma Aldrich, St. Louis, MO, USA). Blood samples were collected within 10 min of completing the measurement. The collected blood was centrifuged at 6000 rpm for 20 min, followed by plasma extraction and cryopreservation (−80 °C) until further analysis. Glutamic oxaloacetic transaminase (GOT), glutamic pyruvic transaminase (GPT), and glucose levels were determined using a blood analyzer (FUJI DRI-CHEM NX 600V, FUJIFILM, Tokyo, Japan). Additionally, cortisol, superoxide dismutase (SOD), and catalase (CAT) levels were analyzed using an ELISA kit (MyBioSource, San Diego, CA, USA).

### 2.7. Whole-Body Composition

To comprehensively analyze the whole-body composition of the experimental fish, three specimens were randomly selected from each tank and promptly frozen and preserved at −80 °C, until further examination. After freeze-drying, the weight of the samples was measured to assess moisture loss, and all proximate analyses were conducted using the freeze-dried material. Moisture content was assessed through a drying process at 135 °C for 24 h, whereas crude ash content was determined after 2 h of heating at 600 °C. The crude fat content was measured using the Soxhlet extraction method (Tecator Soxtec System 1046, Foss Tecator, Hoganas, Sweden), and the crude protein content was determined employing the Kjeldahl Method (Kjeldahl 8100 Analyzer, Foss Tecator, Hoganas, Sweden). The analysis was carried out according to the AOAC [18] procedure.

### 2.8. Gene Analysis

The expression of genes encoding the digestive enzymes was analyzed to determine the digestive capacity. The digestive enzyme genes that were assessed were trypsin (*try*), α-amylase (*amy*) and lipase (*lip*), and the nutrient transporter genes assessed were solute carrier family 7 member 8 (*slc7a8*), sodium/glucose co-transporter member 1 (*sglt1*), Niemann-Pick C1-Like 1 (*npc1l1*) and Glyceraldehyde-3-phosphate dehydrogenase (*gap*). β-actin was used as the reference gene for normalization, following previous studies on digestive gene expression in teleost fish, where it exhibited low variability [21].

Middle gut samples from five randomly selected fish in each tank were collected, promptly frozen, and preserved at −80 °C, until further analysis. RNA extraction from the eel samples was carried out using an RNase mini kit (QIAGEN, Hilden, Germany) following the manufacturer’s protocol. Subsequently, cDNA was synthesized using the M-MLV cDNA Synthesis Kit (enzynomics, Daejoen, Republic of Korea).

Real-time quantitative reverse transcription PCR was employed to analyze the expression of digestion-related genes. Gene-specific primers are detailed in Table 2. The PCR cycling conditions comprised 40 cycles of denaturation at 95 °C for 10 s and annealing and extension at 60 °C for 45 s. Thermal cycling was conducted using the CronoSTAR™ 96 Real-Time PCR System (Takara, Shiga, Japan), and fluorescence detection was performed using TOPrealTM SYBR Green qPCR PreMIX (enzynomics).

The expression levels of the target genes were normalized to an endogenous reference, β-actin, and presented as the subtraction of target CT values from β-actin CT values (ΔCT value). Gene expression between the groups and calibrator was compared by subtracting the calibrator ΔCT values from the target ΔCT values, resulting in a ΔΔCT value. Relative gene expression was determined to calculate the fold difference (2^−ΔΔCT^).

### 2.9. Statistical Analyses

In this study, water quality and growth were assessed at the tank level, with statistical analysis based on the mean values per tank. Specifically, daily water quality values were first averaged per tank before statistical analysis. In contrast, blood and genetic analyses were conducted at the individual fish level, using mean values per eel for statistical evaluation. Statistical analyses were conducted using two-way ANOVA [2 (aquaculture systems: FTS, RAS) × 2 (feed types: paste, pellet)] with the SPSS 24 program (SPSS Inc., Chicago, IL, USA) to assess the main effects and interaction effects for all factors. Prior to conducting the two-way ANOVA, assumptions of normality and homogeneity of variances were verified. Normality was assessed using the Shapiro–Wilk test, and homogeneity of variances was checked using the Levene’s test. All data are expressed as mean ± standard deviation (S.D.). Interaction effects between groups were examined, and the significance of experimental groups was assessed through main effect analysis using the Bonferroni test (*p* < 0.05). Additionally, one-way ANOVA was conducted to analyze single-factor differences in water quality, growth performance, morphological and hematological indices and whole-body compositions. Post hoc testing was conducted using the Duncan’s test (*p* < 0.05). Effect sizes were calculated to determine the magnitude of the observed effects. Additionally, a repeated measures ANOVA was conducted to analyze changes in water quality over time. Mauchly’s test of sphericity was performed, and if the assumption was violated, the Greenhouse–Geisser correction was applied. Statistical analyses were conducted using SPSS, with a significance level set at 0.05.

## 3. Results

### 3.1. Water Quality Analysis

The results of the water quality analysis over 62 days while supplying paste feed and floating extruded pellet to Japanese eels in the FTS and RAS aquaculture systems are presented in Table 3. The water temperature was consistently within the range of 24.4–24.7 °C, DO maintained a range of 6.8–7.1 mg/L, and pH remained within the range of 6.77–6.86, consistent with the predetermined environmental conditions. Significant differences were observed in TAN, NO_2_^−^-N concentrations among experimental groups (*p* < 0.05). The RAS-paste group showed the highest TAN (1.28 ± 0.78 mg/L) and NO_2_^−^-N (0.852 ± 0.435 mg/L) concentrations (*p* < 0.05).

**Figure 2:**
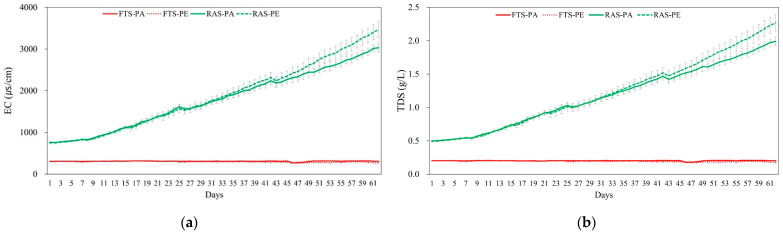
Comparison of (**a**) electric conductivity (EC; μs/cm) and (**b**) concentration of total dissolved solids (TDS; g/L) in Japanese eel (*Anguilla japonica*) fed paste (PA) and floating extruded pellet (PE) in two different systems (FTS, Flow through system; RAS, recirculating aquaculture system) during 62 days of experiment in triplicate.

**Figure 3:**
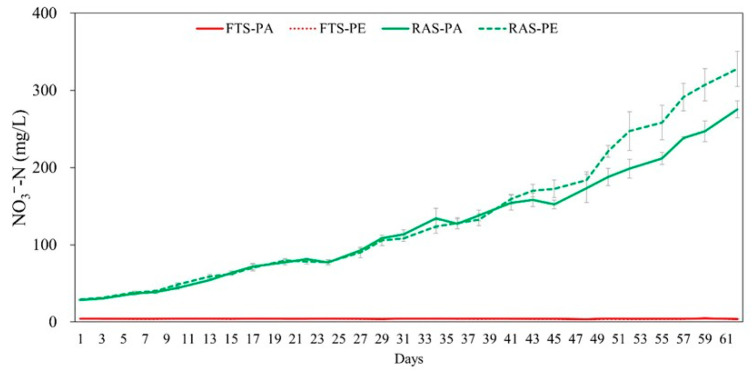
Comparison of nitrate nitrogen (mg/L) concentration in Japanese eel (*Anguilla japonica*) fed paste (PA) and floating extruded pellet (PE) in two different systems (FTS, Flow through system; RAS, recirculating aquaculture system) during 62 days of experiment in triplicate.

Significant interactions between aquaculture systems and feed types were found in TAN and NO_2_^−^-N (*p* < 0.05). In the RAS group, TAN and NO_2_^−^-N were significantly higher than the FTS group, averaging 1.00 ± 0.64 and 0.757 ± 0.464 mg/L (*p* < 0.05). When utilizing paste feed, concentrations resulted in higher TAN (0.859 ± 0.651) and NO_2_^−^-N (0.485 ± 0.502 mg/L), respectively, compared to floating extruded pellet (*p* < 0.05). Electrical conductivity and TDS increased over time in the RAS experimental group (Figure 2). Nitrate nitrogen was maintained at an average of 4.17 ± 0.31 mg/L in the FTS experimental group, but exhibited a steady increase in the RAS experimental group (302 ± 33 mg/L, Figure 3).

According to the results of repeated measures ANOVA (Table 4), time (Days) showed a significant effect on all water quality measurements (*p* < 0.05). Similarly, the interaction between time and treatment groups (Days × Treatment) was significant for all parameters (*p* < 0.05), with the exception of temperature (*p* = 0.071) and DO (*p* = 0.137).

### 3.2. Growth Rate

The growth performance results for eels fed paste or floating extruded pellets, according to the aquaculture system, are presented in Table 5. While the aquaculture system had no significant effect on eel growth (*p* > 0.05), the feed type showed a significant difference (*p* < 0.05). However, no significant interaction effect was observed between the aquaculture system and feed type (*p* > 0.05). Eels fed floating extruded pellet exhibited significantly higher WGR and SGR, at 154 ± 10% and 1.50 ± 0.06%/day, respectively (*p* < 0.05). Throughout the experiment, food intake exhibited a significant difference depending on the aquaculture system and feed type (*p* < 0.05), with higher feed intake observed in the RAS (at 4.16 ± 0.23 kg) and paste groups (4.20 ± 0.25 kg). However, no significant interaction was observed between the aquaculture system and feed type for food intake (*p* = 0.235).

The CF, VSI, and HSI results for eels are presented in Table 6. Significant interactions were found for HSI between aquaculture systems and feed types (*p* < 0.05), whereas no significant interaction effect was observed for CF and VSI (all factors, *p* > 0.05).

### 3.3. Blood Parameters

The results of the eel stress analysis are presented in Table 7. Cortisol and SOD were significantly higher in the FTS-paste group (21.1 ± 9.8 and 150 ± 51 ng/mL, respectively, *p* < 0.05), and GPT was significantly higher in the RAS-paste group (9.80 ± 1.32 U/L, *p* < 0.05) as compared to the other experimental groups. Significant differences were noted for cortisol, SOD, and GPT between the aquaculture systems (*p* < 0.05), whereas the feed type exhibited no significant differences (all factors, *p* > 0.05). Moreover, no significant interaction was identified between the aquaculture system and feed type (all factors, *p* > 0.05). Cortisol and SOD exhibited higher values in the FTS group (18.7 ± 9.7 ng/mL and 129 ± 45 ng/mL, respectively). Conversely, GPT showed higher levels in the RAS group (9.37 ± 1.40 U/L). Glucose, CAT, and GOT showed no significant differences between aquaculture systems (*p* > 0.05).

### 3.4. Whole-Body Composition

The eel whole-body composition results are outlined in Table 8. Crude protein levels were significantly higher in the FTS-pellet (20.1 ± 0.4%) and RAS-pellet (19.7 ± 0.5%) groups compared to the others (*p* < 0.05).

Conversely, the RAS-paste group was significantly higher in crude fat (13.9 ± 0.2%, *p* < 0.05) as compared to the other groups. Moisture content exhibited no significant difference based on the aquaculture system (*p* = 0.189) or feed type (*p* = 0.097). Significant differences were noted in the crude protein and crude fat between the aquaculture systems and feed types (*p* < 0.05). Crude protein displayed higher values in the FTS group (19.5 ± 0.8%) and floating extruded pellet groups (19.9 ± 0.5%), whereas crude fat exhibited higher values in the RAS group (13.6 ± 0.4%) and paste feed group (13.6 ± 0.4%). Two-way ANOVA revealed no significant interaction effect on crude protein (*p* = 0.863), whereas a significant interaction effect was observed in crude fat (*p* < 0.05).

### 3.5. Gene Expression of Digestion-Related Enzymes

**Figure 4:**
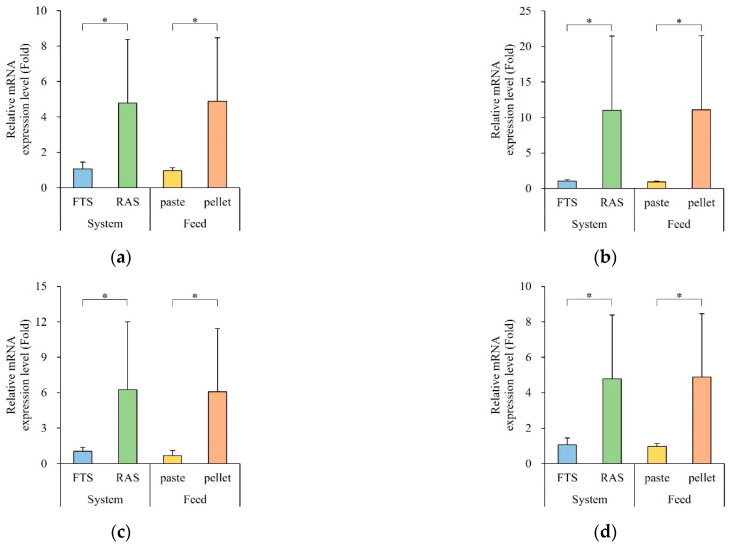

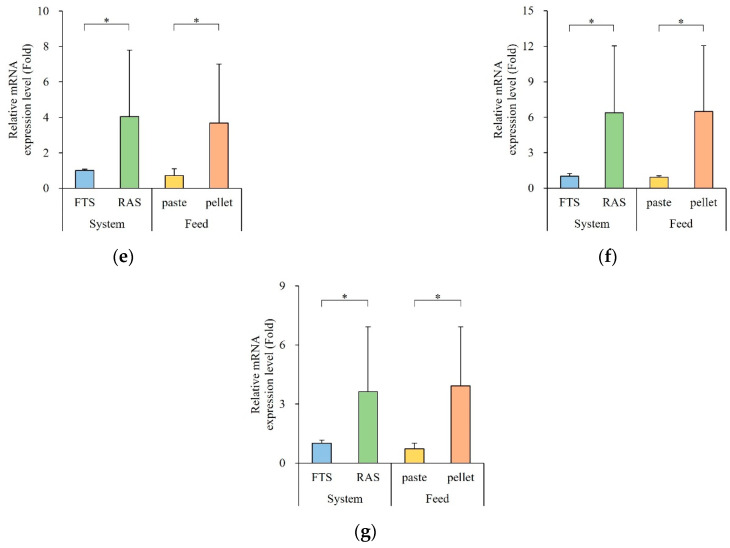
Expression of digestive enzyme and nutrient transporter genes in the gut (n = 5). (**a**) try, (**b**) amy, (**c**) lip, (**d**) slc7a8, (**e**) sglt1, (**f**) npc1l1, and (**g**) gap (try, Trypsin; amy, α-Amylase; lip, Lipase; slc7a8, Solute carrier family 7 member 8; sglt1, Sodium/glucose cotransporter 1; npc1l1, Niemann-Pick C1-LIKE 1; gap; Glyceraldehyde-3-phosphate dehydrogenase). Quantitative real time PCR analysis was performed with equal amounts of total RNA from the gut. β-actin was used as an internal control. * letters indicate significantly different (* *p* < 0.05).

The expression levels of digestion enzyme genes are depicted in Figure 4. Gene expression exhibited significant variations based on the aquaculture system and feed type. Notably, the RAS group showed a significantly higher expression of digestion enzyme and nutrient transporter genes compared to the FTS group (*p* < 0.05), while the floating extruded pellet groups exhibited higher expression levels than the paste groups (*p* < 0.05).

Specifically, the expression level of *try* was 4-times higher in the RAS group (222 ± 2-fold) and 444-times higher in the pellet group (249 ± 2-fold). The expression level of *amy* was 10-times higher in the RAS group (11.0 ± 10.5-fold) and 12-times higher in the pellet group (11.1 ± 10.4-fold). The expression level of *lip* was 6-times higher in the RAS group (6.25 ± 5.76-fold) and 9-times higher in the pellet group (6.08 ± 5.35-fold). Moreover, the expression levels of *slc7a8*, *sglt1*, and *gap* were 4-times higher in the RAS group (4.78 ± 4.78-, 4.05 ± 3.74-, and 3.63 ± 3.28-fold, respectively) and 5-times higher in the pellet group (4.89 ± 3.8-, 3.67 ± 3.33-, and 3.92 ± 3.00-fold, respectively). The expression level of *npc1l1* was 6-times higher in the RAS group (6.38 ± 5.65-fold) and 7-times higher in the pellet group (6.48 ± 5.57-fold).

## 4. Discussion

This study was aimed at validating the feasibility of using floating extruded pellet in RAS for eel cultivation by systematically comparing and analyzing various aspects of eel aquaculture, including water quality, growth, and health, under different feed types (paste and floating extruded pellet) and aquaculture systems (FTS and RAS). The results highlight the importance of suitable feed selection in maintaining optimal RAS conditions, particularly in reducing the impact on water quality and equipment efficiency observed with paste feed use. These comparisons were intended to determine the impact of feed type and aquaculture system on eel performance, ultimately contributing to the optimization of eel aquaculture practices.

### 4.1. Water Quality

Throughout the experimental period, the eel rearing temperature was continuously maintained at 24 °C, aligning with the optimal water temperature range recommended for eel cultivation (22.5 to 26.5 °C) [24,25]. The EC of water, reflective of the concentration and ion charge of dissolved ions, served as an indicator of TDS. The increase in EC was associated with nutrient accumulation in the tank, stemming from low water exchange rates, feed loss, and decomposition of fish waste [26,27]. Notably, the RAS experimental group, characterized by minimal water exchange, exhibited a progressive rise in EC and TDS due to the escalating supply of unfiltered suspended matter alongside the feed. Additionally, when sodium bicarbonate dissolves in water, its dissociated ions facilitate the conduction of electrical current, thereby increasing the electrical conductivity [28].

Our findings further suggest that the accumulation of nitrogen compounds in RAS is significantly influenced by feed type, with paste feed contributing more heavily to TAN and NO_2_^−^-N levels due to its disintegrating characteristics. In RAS, characterized by limited water exchange, effective water treatment for processes, such as nitrification and solids removal, emerged as a critical factor. The design necessitates equilibrium management of parameters, such as oxygen and ammonia, to ensure water quality and productivity [29,30]. To prevent nitrogen loading, primarily in the form of increasing nitrate concentrations, stringent control measures, including ammonia thresholds and appropriate flow rate calculations through mass balance, were employed. The need to optimize biofilter performance and flow rate becomes evident in maintaining suitable water conditions, especially when paste feed is used. Thus, optimizing the biofilter efficiency and proper flow rate are critical in maintaining water quality and safeguarding the experimental fish [31,32]. In addition, pH must also be monitored for the performance of the biofilter. The pH is a key water parameter that impacts the activity of nitrifying bacteria. This, in turn, affects nitrification rates, and indirectly impacts TAN concentrations [33,34]. According to Timmons et al. [35], the recommended pH range for aquaculture systems is between 6.5 and 8.5. In this experiment, we maintained the pH level at an average of 6.8 by adding sodium bicarbonate, ensuring consistency with the pH level of the FTS group to minimize variables.

Across the experimental groups, TAN and NO_2_^−^-N levels were diligently maintained below 2.35 and 2.96 mg/L, respectively. These values adhered to the safe concentrations for TAN and NO_2_^−^-N at pH 7, as suggested by Choe et al. [19]. Elevated levels of TAN and NO_2_^−^-N were observed in the RAS and paste feed groups. Unlike the continuously flowing water in FTS, the closed system in RAS, coupled with the loss-inducing nature of paste feed, led to the accumulation of solids in the water pipes, diminishing the circulation rate of culture water. This phenomenon was confirmed by Obirikorang et al. [36], who demonstrated that lower circulation rates resulted in increased solids and subsequently higher TAN concentrations.

The results of this experiment underscored a tendency for NO_3_^−^-N levels to continue increasing in the RAS group due to non-exchange water conditions. Nitrate oxidation becomes challenging in the biological filtration tank of RAS, where the nitrification process transpires. The prolonged residence time of culture water and a low water exchange rate in RAS contributed to an escalating nitrate concentration [29]. While nitrate toxicity is relatively lower than that of ammonia and nitrite, its continued accumulation could adversely affect the health and survival rates of reared organisms. Effective management strategies, such as water exchange or denitrification devices, are imperative to regulate nitrate concentrations in RAS [37,38,39]. Although the final NO_3_^−^-N concentration of 328 mg/L exceeded the optimal range suggested by Bhatnagar and Devi, it fell below the median lethal concentration of NO_3_^−^-N in freshwater fish, surpassing 1000 mg/L [40]. Thus, the nitrate levels observed in this study were considered sublethal but elevated enough to warrant attention regarding potential physiological impacts. Notably, no abnormal behavior, physiological symptoms, or growth suppression were observed in the eels throughout the experiment, and no clear relationship was found between nitrate concentration and stress or growth responses. The implications of NO_3_^−^-N concentration on eel stress and growth reduction remain understudied, emphasizing the need for additional research to enhance survival rates and maintain optimal culture environments in RAS.

In the RAS experimental tanks, the floating extruded pellet group exhibited lower concentrations of TAN and nitrite, along with higher nitrate levels, indicating more efficient nitrification compared to the paste group. This process, where TAN is sequentially oxidized to nitrite and then to nitrate, led to greater nitrate accumulation in the pellet-fed tanks. Since nitrate is a dominant anion in aquatic systems, its increased concentration contributed to higher EC and TDS [41]. In contrast, the lower nitrification efficiency observed in the paste-fed tanks likely resulted in reduced nitrate production, leading to comparatively lower EC and TDS levels. This finding supports the idea that nitrification efficiency in RAS is lower in tanks receiving paste feed, likely due to its previously discussed limitations, compared to those fed with pellets. These findings highlight how feed type influences nitrification efficiency and subsequent water quality dynamics in RAS.

### 4.2. Growth Rate Analysis

In the 62-day eel culture experiment involving distinct aquaculture systems and feed types, the survival rate exceeded 98% and demonstrated no discernible impact across experimental groups. Notably, the experimental group fed floating extruded pellets exhibited higher TW, WGR, and SGR compared to the paste feed group. Despite the increased feed intake in the paste feed group, a lower growth rate was observed, and was attributed to significant loss inherent in paste feed characteristics [9]. This finding aligns with a prior study on Japanese eel by Kim et al. [10] and an experiment by Höuner et al. [42] involving thin-lip mullet (*Liza ramada*), both indicating superior growth rates in aquaculture systems when using floating pellet feed, corroborating the outcomes of the present study. Due to the difficulty in determining the amount of paste feed loss, the feed intake for the paste feed group in this experiment gives estimated values. However, the lower growth observed for the paste feed group likely reflects lower actual feed intake due to feed disintegration and a lower energy content of the paste feed, which contains less crude fat than the pellet diet. This indicates that paste feed has lower feed efficiency and is less economical than floating extruded pellets. Furthermore, the increased levels of TAN and NO_2_^−^-N observed in the paste feed group, likely caused by the loss-prone nature of the paste feed, may have also contributed to the reduced growth performance observed in this group. However, our study is limited by the fact that the feed intake of the paste feed group is not accurate and pelleted and paste feeds used in the experiment were not iso-energetic. Future experiments should address this limitation by developing and testing iso-energetic paste and pellet feeds for more precise growth comparisons.

VSI and HSI assume crucial roles in elucidating metabolic activities of fish, including digestion, absorption, synthesis and secretion of digestive enzymes, and carbohydrate metabolism [43]. While CF, VSI, and HSI values typically increase with augmented food supply due to the involvement of the intestines and liver in energy storage [44,45,46], this experiment revealed that the higher consumption in the paste feed group did not significantly affect CF, VSI, and HSI values. This observation suggests that the actual intake was lower than the supplied amount because of the high loss rate inherent in paste feed, combined with its lower energy content.

### 4.3. Hematological Analysis

Hematological parameters serve as crucial indicators for evaluating stress response in fish [47,48]. The cortisol range observed in this experiment (5–20 ng/mL) aligns with findings in a study by Amano et al. [49], in which juvenile Japanese eel (*Anguilla japonica)* exhibited comparable cortisol levels (approximately 9–22 ng/mL) across varying rearing densities. Significantly higher cortisol concentrations were detected in the FTS group compared to the RAS group, which may reflect a greater physiological response to environmental fluctuations in FTS, likely attributable to the pronounced water temperature difference between inflow water and rearing water, coupled with temperature fluctuations along the water column [50]. On the other hand, although no significant differences were observed, glucose levels tended to be lower in the FTS group than in the RAS group. Under stressful conditions, fish typically exhibit an initial cortisol response, which subsequently stimulates glucose production as a secondary reaction to provide an energy source for increased metabolic activity [48,49]. However, prolonged exposure to stress may lead to a reduction in glucose levels [51,52].

Similarly, the SOD concentration was relatively higher in the FTS group than in the RAS group, suggesting increased oxidative stress. Environmental stress can lead to the excessive generation of reactive oxygen species, which, if not efficiently neutralized, can cause cellular damage. In response, fish activate antioxidant defense mechanisms, including enzymes such as SOD and CAT, to mitigate oxidative stress effects [48,53,54]. The elevated SOD levels observed in the FTS group may be associated with increased oxidative challenges, likely due to the continuous exposure to environmental fluctuations, particularly temperature variations caused by the inflow of low-temperature groundwater.

Marker of liver function, GPT, witnessed significant differences depending on the aquaculture system, with higher values detected in RAS compared to FTS, aligning with the findings of Zhang et al. [55] in European sea bass (*Dicentrarchus labrax*). These enzymes serve as valuable indicators for evaluating fish health status and detecting potential liver damage under the influence of aquaculture system variations. Sun et al. [56] have reported that increased plasma GPT activity in fish exposed to nitrite could be attributed to the toxic effects of nitroso-compounds, which induce hepatic necrosis. Given that nitrite levels were higher in the RAS group than in the FTS group in this study, the elevated GPT values may be related to prolonged exposure to nitrite concentrations.

Overall, the plasma levels of glucose, GOT, and GPT in this study were comparable to or lower than those reported in a previous study that used a blood chemistry analyzer from the same manufacturer [57]. These results suggest that the values measured here fall within a physiologically acceptable range for Japanese eel (*A. japonica*).

### 4.4. Whole-Body Composition

Fish body composition is intricately affected by feed protein and lipid content, as well as the rearing environment [58,59]. Protein in the feed primarily serves maintenance and tissue protein synthesis, distinct from energy metabolism. Studies have indicated that an excess supply of feed over appropriate intake leads to the conversion of protein to fat because of energy metabolism, resulting in decreased protein and increased fat content stored in the body [46,60,61,62].

In this experiment, the results revealed higher feed intake in the RAS group compared with that in the FTS group, and in paste feed compared with that in the floating extruded pellet. The crude fat content of eels was significantly higher in the RAS and paste feed groups, consistent with the trend observed in feed intake. Conversely, the results for crude protein exhibited an opposite trend to crude fat. Research findings indicate that fat content in the body may vary based on fat content in the feed [61,63]. Despite the higher crude fat content in the pellet feed group, the experiment revealed a significantly higher crude fat content in the paste feed group. This suggests that the fish body composition is not significantly affected by the fat content of the supplied feed. Additional research is warranted to elucidate how the absorption and accumulation of energy, protein, and fat influence body composition.

### 4.5. Expression Levels of Genes Encoding the Digestive Enzymes

Higher expression of digestive enzyme genes was observed in the floating extruded pellet group, indicating improved nutrient breakdown and absorption efficiency. Digestive enzymes involved in nutrient breakdown, such as trypsin (protein), amylase (carbohydrate), and lipase (lipid), a as well as genes associated in nutrient absorption and metabolism, such as *npc1l1* (cholesterol and lipid absorption), *slc7a8* (amino acid transporter), *sglt1* (glucose transporter), and *gap* (glycolysis) [22,64,65,66,67,68,69,70,71], all exhibited increased expression in the pellet-fed group.

In this experiment, the group fed floating extruded pellets exhibited higher growth than the paste feed group, which corresponded with the observed expression levels of digestion-related genes. Digestive enzymes and nutrient transporters in the fish intestine play crucial roles in enhancing nutrient breakdown and absorption, which directly affects their overall growth [70,72,73,74]. The expression levels of digestive enzymes are known to fluctuate according to the protein and lipid content of the feed [68,70]. Although the protein content of the feeds used in this experiment did not differ significantly, the lipid content in the extruded pellet feed was approximately 2% higher. This difference may explain the notably increased expression level of lipase in the pellet-fed group. Furthermore, the lower actual intake in the paste feed group compared with that in the pellet group might have resulted in the higher expression of the digestive enzyme genes, reflecting the higher nutrient intake in the pellet group rather than due to differences in the feed composition.

However, the upregulation of gene expression does not always translate directly into physiological outcomes such as growth, as it can be influenced by factors like post-transcriptional regulation [72]. In this context, the observed expression patterns are better interpreted as an early molecular response to dietary and environmental conditions, rather than a direct indicator of growth performance.

## 5. Conclusions

The results of this study suggest that effectively applying floating extruded pellet feed in RAS may contribute to improved water quality, enhanced system stability, and better growth performance in eels under the given experimental conditions. The higher feed intake and lower feed loss of floating feed likely contributed to the increased growth rate. Additionally, the RAS system appeared to provide a more stable environment for reducing stress levels compared to FTS. Therefore, this study suggests that substituting paste feed with floating extruded pellet feed could be a viable option in eel aquaculture, particularly under similar system conditions. Future studies should validate these results using feeds with identical formulations and develop accurate methods for measuring feed loss in paste feed setups for data reliability. Moreover, future studies should also validate these findings under high-density (≥50 kg/m^3^) and elevated temperature (28–30 °C) conditions to reflect commercial eel farming environments.

## Figures and Tables

**Table 1:** Proximate and ingredient composition of paste and floating extruded pellet feed used in the experiments.

	PA *	PE **
Proximate composition (% dry matter)		
Moisture	5.8	5.4
Crude protein	67.2	67.7
Crude fat	7	9.6
Crude ash	11	10.7
Ingredient composition (% inclusion level)		
Animal protein sources	≥73 (Fish meal, hydrolyzed protein)	≥73 (Fish meal, krill)
Plant protein sources	≤0	≤9 (Soybean meal, gluten)
Cereals	≤23 (Modified starch)	≤12 (Wheat flour, starch)
Lipid sources	≥0	≥5 (Fish oil)
Additives	≥4 (Fermented grain meal, Mono calcium phosphate, lecithin, vitamin premix, mineral premix)	≥1 (Calcium, vitamins, minerals, immune enhancers, amino acids)

PA *, Paste provided by Suhyup, Uiryeong, Republic of Korea; PE **, Pellet provided by Purina, Seongnam, Republic of Korea Based on the ingredient information provided on the product labels.

**Table 2:** Primer sequences of digestion-related genes used in this study.

Genes	Forward (5′ → 3′)	Reverse (5′ → 3′)	Reference
*β-actin*	AATCCACGAGACCACCTTCAACT	TGATCTCTTTCTGCATTCTGTCG	Present study
*try*	CGCTCACTGCTACAAATCTC	CATGATGTCACTGTCCAGGT	[22]
*amy*	ATGGAAGGACGTCCATAGTTC	TGCTAAGTACCGCTCACATTC	[23]
*lip*	CTCCTGACTGGGACAATGAG	GTAGGCTTCGTACGTGTTCC	[22]
*slc7a8*	GATGCTGGTGCACTTCTTCA	CACTGACGGTTGTGTTCCTG	[23]
*sglt1*	GGTCCTCTTCCACGTCCAT	TCTGTATCGCCTGGTCTGG	[22]
*npc1l1*	ATGTCACATCAGGGTCTTCAA	ATGCCATGAATCTTGAGATGA	[22]
*gap*	GCCAGCCAGAACATCATC	GACACGGAAAGCCATACC	Present study

*try*, Trypsin (GenBank accession no. AB070720); *amy*, α-Amylase (GenBank accession no. AB070721); *lip*, Lipase (GenBank accession no. AB070722); *slc7a8*, Solute carrier family 7 member 8; *sglt1*, Sodium/glucose co-transporter member 1; *npc1l1*, Niemann-Pick C1-Like 1; *gap*, Glyceraldehyde-3-phosphate dehydrogenase.

**Table 3:** Water quality of Japanese eel (*Anguilla japonica*) fed paste and floating extruded pellet in different systems for 62 days of experiment.

		Temperature (°C)	DO (mg/L)	pH	TAN (mg/L)	NO_2_^−^-N (mg/L)
One-way ANOVA						
* FTS	Paste	24.4 ± 0.2 ^c^	6.83 ± 0.35 ^b^	6.86 ± 0.12 ^a^	0.530 ± 0.159 ^c^	0.151 ± 0.102 ^c^
	Pellet	24.7 ± 0.2 ^a^	6.80 ± 0.34 ^b^	6.85 ± 0.13 ^a^	0.396 ± 0.108 ^c^	0.146 ± 0.093 ^c^
** RAS	Paste	24.5 ± 0.3 ^b^	6.85 ± 0.41 ^b^	6.81 ± 0.22 ^ab^	1.28 ± 0.78 ^a^	0.852 ± 0.435 ^a^
	Pellet	24.7 ± 0.2 ^a^	7.06 ± 0.30 ^a^	6.77 ± 0.23 ^b^	0.806 ± 0.356 ^b^	0.671 ± 0.386 ^b^
		*p* = 0.00	*p* = 0.00	*p* = 0.032	*p* = 0.00	*p* = 0.00
Two-way ANOVA						
System		*p* = 0.215	*p* = 0.00	*p* = 0.00	*p* = 0.00	*p* = 0.00
	FTS	24.6 ± 0.3	6.81 ± 0.40	6.86 ± 0.14	0.463 ± 0.169	0.141 ± 0.105
	RAS	24.6 ± 0.4	6.96 ± 0.43	6.79 ± 0.27	1.00 ± 0.64	0.757 ± 0.464
Feed		*p* = 0.00	*p* = 0.005	*p* = 0.058	*p* = 0.00	*p* = 0.025
	Paste	24.5 ± 0.4	6.84 ± 0.45	6.84 ± 0.20	0.859 ± 0.651	0.485 ± 0.502
	Pellet	24.7 ± 0.3	6.93 ± 0.39	6.81 ± 0.23	0.599 ± 0.344	0.410 ± 0.401
Interaction		*p* = 0.021	*p* = 0.00	*p* = 0.415	*p* = 0.005	*p* = 0.011

* FTS, Flow through system; ** RAS, Recirculating aquaculture system; a, b, c letters indicate significantly different (*p* < 0.05)

**Table 4:** Repeated measures ANOVA results for water quality measurements for 62 days of experiment.

Parameter	Days (*p*-Value)	Days × Treatment (*p*-Value)	Partial η^2^ (Time)	Partial η^2^ (Interaction)
Temperature	0.005	0.071	0.293	0.382
DO	0.000	0.137	0.749	0.436
pH	0.000	0.000	0.745	0.834
EC	0.000	0.000	0.994	0.982
TDS	0.000	0.000	0.994	0.992
TAN	0.000	0.000	0.732	0.768
NO_2_^−^-N	0.000	0.009	0.733	0.621
NO_3_^−^-N	0.000	0.000	0.988	0.986

**Table 5:** Growth performance of Japanese eel (*Anguilla japonica*) fed paste and floating extruded pellet in two different systems for 62 days of experiment.

		Final BW (g)	Final TW (kg)	Final Density (kg/m^3^)	^1^ WGR (%)	^2^ SGR (%/day)	^3^ Survival Rate (%)	Feed Intake (kg)
One-way ANOVA								
* FTS	Paste	75.4 ± 4.8	4.67 ± 0.30 ^b^	11.7 ± 0.7 ^b^	134 ± 17	1.37 ± 0.11	98.9 ± 0.9	4.08 ± 0.32 ^a^
	Pellet	78.9 ± 3.4	4.91 ± 0.11 ^ab^	12.3 ± 0.3 ^ab^	148 ± 9	1.46 ± 0.06	98.4 ± 1.6	3.46 ± 0.62 ^b^
** RAS	Paste	75.4 ± 5.1	4.62 ± 0.19 ^b^	11.6 ± 0.5 ^b^	135 ± 12	1.38 ± 0.08	98.9 ± 1.9	4.33 ± 0.08 ^a^
	Pellet	83.3 ± 1.9	5.17 ± 0.20 ^a^	12.9 ± 0.5 ^a^	159 ± 9	1.54 ± 0.05	100 ± 0	4.00 ± 0.21 ^a^
		*p* = 0.119	*p* = 0.045	*p* = 0.045	*p* = 0.092	*p* = 0.111	*p* = 0.541	*p* = 0.04
Two-way ANOVA								
System		*p* = 0.358	*p* = 0.435	*p* = 0.435	*p* = 0.410	*p* = 0.399	*p* = 0.336	*p* = 0.009
	FTS	77.1 ± 4.2	4.79 ± 0.24	12.0 ± 0.6	141 ± 14	1.42 ± 0.10	98.7 ± 1.2	3.77 ± 0.40
	RAS	79.4 ± 5.5	4.89 ± 0.35	12.2 ± 0.9	147 ± 16	1.46 ± 0.11	99.5 ± 1.4	4.16 ± 0.23
Feed		*p* = 0.038	*p* = 0.012	*p* = 0.012	*p* = 0.023	*p* = 0.028	*p* = 0.721	*p* = 0.003
	Paste	75.4 ± 4.4	4.65 ± 0.23	11.6 ± 0.6	135 ± 13	1.37 ± 0.09	98.9 ± 1.3	4.20 ± 0.25
	Pellet	81.1 ± 3.4	5.04 ± 0.20	12.6 ± 0.5	154 ± 10	1.5 ± 0.06	99.2 ± 1.3	3.73 ± 0.33
Interaction		*p* = 0.367	*p* = 0.243	*p* = 0.243	*p* = 0.489	*p* = 0.561	*p* = 0.318	*p* = 0.235

* FTS, Flow through system; ** RAS, Recirculating aquaculture system; ^1^ WGR, Weight gain rate (%) = [Final weight (g) − initial weight (g) × 100]/initial weight (g); ^2^ SGR, Specific growth rate (%/day) = [Log_e_ (final weight (g)) − Log_e_ (initial weight (g))] × 100/days; ^3^ Survival rate (%) = (Final number of individuals/initial number of individuals) × 100; Values (mean ± SD of values for replicates) in the same column sharing the same superscript letter are not significantly different (*p* > 0.05). a, b letters indicate significantly different (*p* < 0.05).

**Table 6:** Comparison of morphological indices of Japanese eel (*Anguilla japonica*) fed paste and floating extruded pellet in two different systems for 62 days of experiment.

		^1^ CF	^2^ VSI (%)	^3^ HSI (%)
One-way ANOVA				
* FTS	Paste	0.13 ± 0.01	5.41 ± 0.23	1.67 ± 0.07 ^a^
	Pellet	0.13 ± 0.00	5.33 ± 0.17	1.33 ± 0.13 ^b^
** RAS	Paste	0.13 ± 0.01	5.29 ± 0.41	1.45 ± 0.14 ^ab^
	Pellet	0.13 ± 0.01	5.95 ± 0.30	1.60 ± 0.10 ^a^
		*p* = 0.363	*p* = 0.078	*p* = 0.027
Two-way ANOVA				
System		*p* = 0.580	*p* = 0.175	*p* = 0.699
	FTS	0.13 ± 0.00	5.37 ± 0.18	1.50 ± 0.21
	RAS	0.13 ± 0.01	5.62 ± 0.48	1.53 ± 0.14
Feed		*p* = 0.580	*p* = 0.123	*p* = 0.171
	Paste	0.13 ± 0.01	5.35 ± 0.30	1.56 ± 0.16
	Pellet	0.13 ± 0.00	5.64 ± 0.40	1.46 ± 0.18
Interaction		*p* = 0.122	*p* = 0.061	*p* = 0.006

* FTS, Flow through system; ** RAS, Recirculating aquaculture system; ^1^ CF (Condition factor) = [wet weight (g)/(total length (cm))3] × 100; ^2^ VSI (Viscerosomatic index) (%) = [wet weight of viscera (g)/wet weight (g)] × 100; ^3^ HSI (Hepatosomatic index) (%) = [wet weight of liver (g)/wet weight (g)] × 100; Values (mean ± SD of values for replicates) in the same column sharing the same superscript letter are not significantly different (*p* > 0.05). a, b letters indicate significantly different (*p* < 0.05).

**Table 7:** Comparison of hematological indices of Japanese eel (*Anguilla japonica*) fed paste and floating extruded pellet in two different systems for 62 days of experiment.

		Cortisol (ng/mL)	GLU (md/dL)	SOD (ng/mL)	CAT (U/mL)	GOT (U/L)	GPT (U/L)
One-way ANOVA							
* FTS	Paste	21.1 ± 9.8 ^a^	114 ± 19	150 ± 51 ^a^	44.6 ± 12.0	73.6 ± 18.2	7.73 ± 0.80 ^c^
	Pellet	16.3 ± 9.5 ^ab^	121 ± 29	107 ± 25 ^b^	36.1 ± 18.3	64.6 ± 25.6	7.73 ± 1.03 ^c^
** RAS	Paste	12.1 ± 6.5 ^bc^	127 ± 32	85.2 ± 24 ^b^	41.9 ± 13.5	59.4 ± 45.2	9.80 ± 1.32 ^a^
	Pellet	7.92 ± 5.6 ^c^	130 ± 53	82.8 ± 29 ^b^	49.5 ± 13.9	54.3 ± 30.8	8.93 ± 1.39 ^b^
		*p* = 0.011	*p* = 0.594	*p* = 0.001	*p* = 0.294	*p* = 0.391	*p* = 0.00
Two-way ANOVA							
System		*p* = 0.003	*p* = 0.213	*p* = 0.001	*p* = 0.290	*p* = 0.139	*p* = 0.00
	FTS	18.7 ± 9.7	117 ± 24	129 ± 45	40.1 ± 15.8	69.1 ± 22.3	7.73 ± 0.91
	RAS	10.0 ± 6.3	129 ± 43	84.0 ± 25.6	45.7 ± 13.9	56.9 ± 38.1	9.37 ± 1.40
Feed		*p* = 0.103	*p* = 0.597	*p* = 0.067	*p* = 0.938	*p* = 0.392	*p* = 0.153
	Paste	16.6 ± 9.3	121 ± 27	120 ± 52	43.2 ± 12.5	66.5 ± 34.6	8.77 ± 1.50
	Pellet	12.1 ± 8.7	125 ± 42	95.8 ± 29.1	42.8 ± 17.2	59.5 ± 28.3	8.33 ± 1.35
Interaction		*p* = 0.914	*p* = 0.839	*p* = 0.099	*p* = 0.116	*p* = 0.810	*p* = 0.153

* FTS, Flow through system; ** RAS, Recirculating aquaculture system; Values (mean ± SD of values for replicates) in the same column sharing the same superscript letters are not significantly different (*p* > 0.05). a, b, c letters indicate significantly different (*p* < 0.05).

**Table 8:** Comparison of whole-body composition of Japanese eel (*Anguilla japonica*) fed paste and floating extruded pellet in different systems for 62 days of experiment.

		Moisture (%)	Crude Protein (%)	Crude Fat (%)	Crude Ash (%)
One-way ANOVA					
* FTS	Paste	65.1 ± 0.2	18.8 ± 0.1 ^b^	13.2 ± 0.3 ^b^	2.50 ± 0.09
	Pellet	65.0 ± 0.2	20.1 ± 0.4 ^a^	11.9 ± 0.2 ^c^	2.48 ± 0.21
** RAS	Paste	65.0 ± 0.4	18.3 ± 0.2 ^b^	13.9 ± 0.2 ^a^	2.41 ± 0.05
	Pellet	64.6 ± 0.3	19.7 ± 0.5 ^a^	13.2 ± 0.3 ^b^	2.21 ± 0.13
		*p* = 0.184	*p* = 0.00	*p* = 0.00	*p* = 0.092
Two-way ANOVA					
System		*p* = 0.189	*p* = 0.0028	*p* = 0.00	*p* = 0.045
	FTS	65.1 ± 0.2	19.5 ± 0.8	12.6 ± 0.8	2.49 ± 0.14
	RAS	64.8 ± 0.4	19.0 ± 0.8	13.6 ± 0.4	2.31 ± 0.14
Feed		*p* = 0.097	*p* = 0.00	*p* = 0.00	*p* = 0.180
	Paste	65.1 ± 0.3	18.5 ± 0.3	13.6 ± 0.4	2.46 ± 0.08
	Pellet	64.8 ± 0.3	19.9 ± 0.5	12.6 ± 0.7	2.34 ± 0.22
Interaction		*p* = 0.462	*p* = 0.863	*p* = 0.049	*p* = 0.277

* FTS, Flow through system; ** RAS, Recirculating aquaculture system; Values (mean ± SD of values for replicates) in the same column sharing the same superscript letters are not significantly different (*p* > 0.05). a, b, c letters indicate significantly different (*p* < 0.05).

## Data Availability

Then data underlying this article are available in Figshare (https://doi.org/10.6084/m9.figshare.29557856, accessed on 14 July 2025).
